# Derivation of some contemporary scales to measure adolescent risk-taking in Canada

**DOI:** 10.1007/s00038-017-1046-6

**Published:** 2017-10-25

**Authors:** Jonathan L. Kwong, Don A. Klinger, Ian Janssen, William Pickett

**Affiliations:** 1 Department of Public Health Sciences, Queen’s University, Caruthers Hall, 2nd Floor, Kingston, ON K7L 3N6 Canada; 2 Faculty of Education, Queen’s University, Kingston, ON Canada; 3 School of Kinesiology and Health Studies, Queen’s University, Kingston, ON Canada; 4 Department of Emergency Medicine, Queen’s University, Kingston, ON Canada

**Keywords:** Adolescent, Risk behaviour, Problem Behaviour Theory

## Abstract

**Objectives:**

To derive a contemporary series of composite indicators of adolescent risk-taking, inspired by the US CDC Framework and Problem Behaviour Theory.

**Methods:**

Factor analyses were performed on 28-risk behaviours in a nationally representative sample of 30,096 Grades 6–10 students from the 2014 Canadian Health Behaviour in School-aged Children study.

**Results:**

Three composite indicators emerged from our analysis: (1) Overt Risk-Taking (i.e., substance use, caffeinated energy drink consumption, fighting, and risky sexual behaviour), (2) Aversion to a Healthy Lifestyle (i.e., physical inactivity and low fruit and vegetable consumption), and (3) Screen Time Syndrome (i.e., abnormally high screen time use combined with unhealthy snacking). These three composite indicators of risk-taking were observed consistently with strong psychometric properties across different grade groups (6–8, 9–10).

**Conclusions:**

The three composite indicators of adolescent risk-taking each draw from multiple domains within the CDC framework, and support a novel, empirically directed approach of conceptualizing multiple risk behaviours among adolescents. The measures also highlight the breadth and diversity of risk behaviour engagement among Canadian adolescents. Research and preventive interventions should simultaneously consider the related behaviours within each of these composite indicators.

## Introduction

Adolescent risk-taking behaviours are well-established causes of illness and injury (Turner et al. 2004). ‘Problem Behaviour Theory’ is foundational to modern adolescent risk research, and suggests that adolescents develop and exhibit risk-taking behaviours in related groups based on a variety of upstream determinants (called risk factors) (Jessor 1991, 2014). Such risk factors lead to the development of multiple risk behaviours that cluster together in predictable patterns within populations of young people (Pickett et al. 2002; De La Haye et al. 2014; Pfortner et al. 2015). Research and ongoing surveillance efforts in the field of adolescent risk-taking should, therefore, focus on these behaviours, both individually and in composite, to inform prevention efforts aimed specifically at adolescents. While other studies consider “risk behaviours” in the context of intent or motivation (Pérez Fuentes et al. 2016), this study uses the term to define the true action itself that poses potential harm to adolescents (e.g., Pickett et al. 2002; Riesch et al. 2013).

Over 2 decades ago, the US Centers of Disease Control and Prevention (CDC) created a risk behaviour framework that classified adolescent risk behaviours using evidence derived from the US Youth Risk Behaviour Survey (Kann et al. 2016). This long-standing framework categorizes risk behaviours across six domains that are associated with leading causes of morbidity and mortality among American youths: tobacco use, alcohol and illicit substance use, high-risk sexual behaviour, injury-prone and violent behaviour, unhealthy dietary patterns, and physical inactivity (Kann et al. 2016). These domains, informed by peer-reviewed literature (Brener et al. 2004), were established by consensus and are regularly updated to include emergent types of adolescent risk behaviours. Although this framework was initially developed for surveillance and policy development, it is probably the most frequently applied tool for population-based and clinical activities because of its broad consideration of varying behaviours associated with adolescent health.

There is a rich literature available that describes inter-relationships between adolescents’ risk behaviours and their potential effects on a variety of negative health outcomes (Yarber and Parrillo 1992; Lytle 2002; Schane et al. 2010; Spring et al. 2012; Thompson et al. 2014); however, such studies rarely consider the full complement of behaviours outlined within the CDC framework. This is particularly true in our own country of Canada. As a consequence, there may be an incomplete conceptual understanding of contemporary adolescent risk-taking behaviour and how such behaviours tend to develop and occur concurrently. For example, a large body of literature on adolescent risk-taking focuses on behaviours found in the stereotypical delinquent adolescent (i.e., the CDC domains of: alcohol and illicit substance use, tobacco use, and high-risk sexual behaviours) (Lindberg et al. 1995; Turner et al. 2004). Although these behaviours are suggestive of a high-risk lifestyle, they may also be related conceptually (and mathematically) to risk behaviours found in other domains within the CDC framework. In addition, there may be new risk behaviours within the CDC domains reflective of more contemporary patterns [e.g., e-cigarettes in tobacco use, and caffeinated energy drinks in alcohol and illicit substance use (Seifert et al. 2011; Goniewicz et al. 2016)], that are not included in these traditionally defined risk behaviour clusters. Behaviours from those and other domains in the CDC framework may be inter-related in different ways that reveal new patterns of risk behaviour. Finally, it is possible that adolescents in Canada engaged in risk-taking in ways that are unique from their American counterparts, and most of the existing empirical research in this field has been concentrated in the United States (Basen-Engquist et al. 1996; Riesch et al. 2013).

We had the opportunity, through analyses that involved both exploratory and confirmatory methods and a large population-based study of Canadians adolescents (Currie et al. 2012), to perform a contemporary analysis of adolescent risk-taking in Canada. Our objective was to explore inter-relationships between contemporary expressions of adolescent risk-taking, yet inspired by the long-standing CDC framework, and as a result, to create and validate new composite indicators of adolescent risk behaviours in a Canadian adolescent population. Our hope was that this would provide valuable new information in support of preventive initiatives in our country, and perhaps elsewhere.

## Methods

### Study base and sampling

Our study was based on Canadian records (*N* = 30,096) from the Health Behaviour in School-aged Children study (HBSC), a World Health Organization collaborative cross-national study (Currie et al. 2012). Cycle 7 of the Canadian HBSC evaluated health outcomes, attitudes, and behaviours using a confidential questionnaire administered to students from 377 schools during the 2013–14 academic year. The Canadian HBSC followed an international sampling protocol. Classes within selected schools were selected for participation using a weighted probability technique to ensure proportional representation based on the 10 Canadian provinces and three territories and the following demographic characteristics: urban–rural geographic location, language of instruction, religion, and community size. The target age range of students was 11–15 years, which typically corresponds to Grades 6–10 in Canada (Freeman et al. 2016). Grades 6–8 students were given a condensed survey that omitted questions of a more sensitive-nature (i.e., illicit drug use and sexual behaviour). Students enrolled in private, special needs, on-reserve, or faith schools (other than publicly funded Roman Catholic Schools) were not included; they represent < 7% of the Canadian student population in this age range (Van Pelt et al. 2015). Survey weights were applied to ensure that the sample was generalizable to the national population. Additional details on the HBSC study can be found in the 2014 Canadian HBSC report (Freeman et al. 2016).

### Measures of risk behaviour

As per existing precedents, we defined risk behaviours as “voluntary behaviours having known health consequences that can threaten an individual’s successful physical and/or psychosocial development”, acknowledging that risk behaviours can also be part of “normal adolescent development” (Jessor 1991). All risk behaviours that met this definition and were measured in Cycle 7 of the Canadian HBSC were identified. We then categorized each identified risk behaviour (28 identified in total) according to the six domains of risk as outlined in the CDC framework (Kann et al. 2016). To standardize our approach to classification and subsequent factor analysis for categorical variables, we re-coded each of the 28 items into three broad categories based on level of behavioural engagement and group size: low (no or minimal engagement in the risk behaviour), medium, and high (extensive engagement) (see Table 1). A combination of current and lifetime exposure to risk behaviours was studied. This was done intentionally to capture a student’s propensity to engage in certain behaviours earlier in life that might lead to subsequent engagement into different behaviours.

**Table 1:** Initial set of risk behaviours from the HBSC used for exploratory factor analysis (Canada 2014)

Initial set of risk behaviours	None/minimal engagement	Moderate engagement	Frequent engagement
CDC domain 1: smoking cigarettes			
Number of days they smoked cigarettes in their life	Never	1–29 days	30 + days
Alternative tobacco products (e.g., e-cigarette, flavored tobacco…)^b^	Never used any	Used one once or more	Used several once or more
CDC domain 2: alcohol and illicit substance use			
Frequency of alcohol consumption (e.g., beer, wine, cider…)^b^	Never drank any	Rarely	Every month–every day
Number of drinks per typical event	Never drank	Less than 1—one drink	2 + drinks
Number of times they got drunk in their life^a^	Never	Once	2 + times
Frequency of binge drinking in the last year^a^	Never drank-never binged	Less than or once a month	2–3 times a month-daily
Number of days they used cannabis in their life^a^	Never	1–5 days	6 + days
Number of times they used hard drugs (e.g., ecstasy, solvents, pain medication…)^ab^	Never used any	Used one once	Used several once or more
CDC domain 3: high-risk sexual behaviours			
Lifetime sexual history and use of contraceptives^ab^	Never had sex	Had sex using contraception	Sex without contraception
CDC domain 4: high-risk manifest behaviours			
Number of times they got into a fight in the last year	No fights	Once	2 + times
Frequency of personal bullying behaviours on others	No bullying	Once-3 times a month	Once a week or more
Frequency of helmet use while riding a bicycle	Always^c^	Sometimes-most of the time	Never
Frequency of helmet use while in an off-road vehicle	Always^c^	Sometimes-most of the time	Never
CDC domain 5: unhealthy dietary pattern			
Frequency of sugar-sweetened soda consumption in a typical week	Never-once a week	2–4 times a week	5–6 times a week or more
Frequency of chip consumption in a typical week	Never-once a week	2–4 times a week	5–6 times a week or more
Frequency of sweet/candy consumption in a typical week	Never-once a week	2–4 times a week	5–6 times a week or more
Frequency of caffeinated energy drink consumption in a typical week	Never	Less than or once a week	2–4 times a week or more
Frequency of fruit consumption in a typical week	Once or more a day	2–6 times a week	Never-once a week
Frequency of vegetable consumption in a typical week	Once or more a day	2–6 times a week	Never-once a week
Frequency of orange vegetable consumption in a typical week	Once or more a day	2–6 times a week	Never-once a week
CDC domain 6: physical inactivity			
Hours watching TV or videos on a typical day^b^	None-1.5 h	1.5–3 h	3 + hours
Hours playing video games on a typical day^b^	None-1.5 h	1.5–3 h	3 + hours
Hours using a computer on a typical day^b^	None-1.5 h	1.5–3 h	3 + hours
Hours playing outdoors outside of school hours^b^	3 + hours	1–3 h	None-1 h
Participation in organized sports^b^	Both individual and team sport	Either individual or team sport	No participation
Active travel to school (including duration)^b^	Walk/bike 5 min or more	Walk/bike less than 5 min	Not walking or bicycling
Hours participating in physical education at school on a typical week	4–7 h	2–3 h	None-1 h
Hours of exercise outside of school hours	4–7 times a week	2–3 times a week	Never-once a week

Coding of relative risk for each of the risk behaviours is also included. Coding within each level of risk may represent an aggregate of multiple questionnaire options

*HBSC* Health Behaviour in School-aged Children Study, *CDC* Centers for Disease Control and Prevention

^a^Grades 6–8 students are not asked these questions in the HBSC study

^b^Denote risk behaviours that are a composite measure combining multiple HBSC study questionnaire items

^c^This category also includes students who did not ride a bicycle or motor vehicle

### Statistical analyses

Latent risk constructs were identified from the list of risk behaviours and then validated using exploratory and confirmatory factor analyses, respectively (Kline 2013). A split-sampling method was followed, with the study sample randomly divided in half using a simple random sampling technique (equal probability without replacement). Separate exploratory and confirmatory factor analyses were then performed for each of the two grade groups (6–8, 9–10) due to the differences in the available measures of risk behaviours in the two groups. Common factors in the exploratory analyses were extracted using iterated principal axis factoring with promax rotation. Factor loadings below 0.30 were suppressed (Kline 1994). Factor interpretability, scree plots, and parallel analyses (Kabacoff 2003) were used to specify the number of factors to include in the final model (Fabrigar et al. 1999). Confirmatory factor analysis using maximum-likelihood estimation was used with the second group in an attempt to validate the common factor structure. Root-mean-squared error of approximation (RMSEA), standardized root-mean-square residuals (SRMR), and adjusted goodness-of-fit index (AGFI) were used to evaluate model fit (Hooper et al. 2008). Intraclass correlations were calculated separately for all risk behaviours included in the final models to assess for clustering at the school level. Direct correlations and correlations corrected for attenuation (Schmitt 1996) were calculated between identified subscales, and McDonald’s omega was calculated to assess the internal consistency (reliability) for each subscale (Zhang and Yuan 2015). All analyses in this study considered sample weights and were conducted in SAS (Version 9.4, SAS Institute, Cary, NC). McDonald’s omega values were calculated using R (Version 3.4.1, R Foundation for Statistical Computing, Vienna, Austria).

## Results

### Sample population

Of the 30,096 responses available for study, 13,806 were in Grades 9–10 and 16,290 were in Grades 6–8. The proportion of students identified as being in the high-risk category for each of the risk behaviours included in the final model can be found in Table 2.

**Table 2:** Students in each risk level for all risk behaviours in the final exploratory model (Canada 2014)

Final set of risk behaviours	Frequency of risk behaviour engagement						Missing
	None/minimal		Moderate		Frequent		
	No.	Row%	No.	Row%	No.	Row%	
Grades 9–10 students							
CDC domain 1: smoking cigarettes							
Lifetime smoking history	10,682	(80.7)	1759	(13.3)	798	(6.0)	584
Use of alternative tobacco products	9784	(74.1)	1896	(14.4)	1519	(11.5)	624
CDC domain 2: alcohol and illicit substance use							
Frequency of alcohol consumption	5437	(40.8)	4192	(31.5)	3701	(27.8)	492
Number of drinks per typical event	6022	(45.8)	2704	(20.6)	4409	(33.6)	688
Lifetime drunkenness history	8676	(65.3)	3139	(23.6)	1465	(11.0)	543
Binge drinking	8452	(66.1)	2968	(23.2)	1363	(10.7)	1040
Illicit drug use	10,256	(77.7)	594	(4.5)	2351	(17.8)	622
Lifetime cannabis use	10,082	(76.5)	793	(6.0)	2303	(17.5)	646
CDC domain 3: high-risk sexual behaviours							
Sex and contraceptive use	7503	(77.7)	1828	(18.9)	329	(3.4)	4164
CDC domain 4: high-risk manifest behaviours							
Physical fighting	10,199	(75.7)	1558	(11.6)	1715	(12.7)	352
Non-helmet use on a bicycle	4961	(39.4)	3025	(24.1)	4593	(36.5)	1244
CDC domain 5: unhealthy dietary pattern							
Sweet consumption	4601	(34.5)	4510	(33.9)	4211	(31.6)	502
Sugar-sweetened soda consumption	7868	(58.0)	2943	(21.7)	2747	(20.3)	264
Chip consumption	9508	(70.3)	2646	(19.6)	1374	(10.2)	295
Caffeinated energy drink consumption	9921	(72.5)	2848	(20.8)	911	(6.7)	143
Low fruit consumption	6234	(45.5)	5928	(43.3)	1542	(11.3)	119
Low vegetable consumption	6042	(44.4)	5867	(43.1)	1695	(12.5)	219
Low orange vegetable consumption	2096	(15.4)	6174	(45.3)	5351	(39.3)	203
CDC domain 6: physical inactivity							
Watching TV or videos	3619	(28.6)	3885	(30.7)	5151	(40.7)	1168
Playing video games	5282	(41.8)	2599	(20.6)	4750	(37.6)	1193
Using the computer	3847	(30.7)	2603	(20.8)	6073	(48.5)	1301
Outdoor play	3184	(25.5)	5086	(40.8)	4199	(33.7)	1355
Organized sport	4127	(31.3)	5341	(40.5)	3730	(28.3)	626
Outdoor exercise	5605	(42.2)	3747	(28.2)	3926	(29.6)	547
Grades 6–8 students							
CDC domain 1: smoking cigarettes							
Lifetime smoking history	14,730	(94.6)	626	(4.0)	216	(1.4)	723
Use of alternative tobacco products	14,342	(92.5)	745	(4.8)	417	(2.7)	790
CDC domain 2: alcohol and illicit substance use							
Frequency of alcohol consumption	11,050	(70.7)	3278	(21.0)	1293	(8.3)	673
Lifetime drunkenness history	14,290	(91.9)	1046	(6.7)	212	(1.4)	746
CDC domain 4: high-risk manifest behaviours							
Physical fighting	10,717	(68.2)	2285	(14.5)	2715	(17.3)	578
Non-helmet use on a bicycle	7576	(48.6)	4382	(29.3)	3320	(22.2)	1316
CDC domain 5: unhealthy dietary pattern							
Sweet consumption	6339	(40.9)	4666	(30.1)	4489	(29.0)	801
Sugar-sweetened soda consumption	10,550	(66.4)	3040	(19.1)	2297	(11.5)	408
Chip consumption	11,569	(72.9)	2693	(17.0)	1612	(10.2)	420
Caffeinated energy drink consumption	13,285	(82.8)	2153	(13.4)	601	(3.7)	255
Low fruit consumption	8557	(53.2)	6189	(38.5)	1345	(8.4)	203
Low vegetable consumption	7674	(48.4)	6277	(39.6)	1901	(12.0)	443
Low orange vegetable consumption	2959	(18.5)	7131	(44.7)	5868	(36.8)	336
CDC domain 6: physical inactivity							
Watching TV or videos	5449	(36.4)	4463	(29.8)	5043	(33.7)	1338
Playing video games	6769	(45.3)	3473	(23.3)	4696	(31.4)	1357
Using the computer	7161	(48.2)	3035	(20.4)	4668	(31.4)	1430
Outdoor play	4572	(30.8)	6249	(42.1)	4008	(27.0)	1464
Organized sport	5377	(34.7)	6702	(43.2)	3427	(22.1)	788
Outdoor exercise	8020	(50.3)	4331	(27.1)	3607	(22.6)	337

Row percentages do not take missing values into consideration and may not add to 100% due to rounding

*CDC* Centers for Disease Control and Prevention

### Exploratory and confirmatory factor analysis

After consideration of the available 28 risk behaviours, a three-factor solution emerged from the exploratory factor analyses within both grade groups. The final model in both grade groups had an independent cluster solution. Eigenvalues for all common factors in the models were above the 90% confidence intervals from the parallel analyses, suggesting that variances explained by the factors were better than a chance finding. Final eigenvalues for the Grades 9–10 model were 5.08, 2.01, and 1.13 (*N* = 3594). For Grades 6–8 students, findings were consistent; the final eigenvalues were 3.06, 1.65, and 1.08 (*N* = 5586). Only students with responses to all risk behaviours were included in the factor analyses. Given the reduced sample sizes used for the final exploratory factor analyses due to missing responses, sensitivity analyses using full information maximum-likelihood imputation were performed, and no significant changes to factor structure, eigenvalues, or factor loadings were identified.

Based on similar results from the exploratory analyses for both grade groups, the three risk behaviour categories were labeled together based on a general conceptual understanding of the behaviours that emerged from their respective exploratory factor analyses (Tables 3, 4). The first common factor showed behaviours associated with substance use and externalizing risk-taking such as fighting, non-helmet use while riding on a bicycle, and risky sexual behaviour. We called this category ‘*Overt Risk Taking*’. The second factor identified behaviours associated with low consumption of nutritious food (such as fruits and vegetables) and low participation in different forms of moderate-to-vigorous physical activity (such as organized sports and free play). Because of the omission of behaviours associated with healthy, balanced lifestyles, we called this category ‘*Aversion to a Healthy Lifestyle*’. The third factor grouped sedentary screen time activities together with unhealthy snacking behaviours (i.e., potato chip and soda consumption)—we called this category the ‘*Screen Time Syndrome*’. Cronbach’s alpha values calculated for each of the risk behaviour categories in both grade groups were all above 0.60 suggesting acceptable levels of internal consistency.

**Table 3:** Exploratory factor analysis on risk behaviours considering all domains in the US Centers for Disease Control and Prevention risk framework in Grades 9–10 students (*N* = 3594) (Canada 2014)

Risk behaviours	Factor 1 Overt Risk Taking	Factor 2 Aversion to a Healthy Lifestyle	Factor 3 Screen Time Syndrome	Intraclass correlation
CDC domain 1: smoking cigarettes				
Lifetime smoking history	0.65			0.062
Use of alternative tobacco products	0.71			0.048
CDC domain 2: alcohol and illicit substance use				
Frequency of alcohol consumption	0.76			0.080
Number of drinks per typical event	0.80			0.096
Lifetime drunkenness history	0.85			0.065
Binge drinking	0.81			0.061
Illicit drug use	0.33			0.084
Lifetime cannabis use	0.73			0.050
CDC domain 3: high-risk sexual behaviours				
Sex and contraceptive use	0.46			0.045
CDC domain 4: high-risk manifest behaviours				
Non-helmet use on a bicycle	0.32			0.077
CDC domain 5: unhealthy dietary pattern				
Caffeinated energy drink consumption	0.37			0.022
CDC domain 5: unhealthy dietary pattern				
Fruit consumption		0.63		0.040
Vegetable consumption		0.56		0.039
Orange vegetable consumption		0.56		0.020
CDC domain 6: physical inactivity				
Duration of outdoor play		0.39		0.041
Participation in organized sports		0.48		0.024
Frequency of exercise outside school hours		0.47		0.026
CDC domain 5: unhealthy dietary pattern				
Sweet consumption			0.39	0.011
Sugar-sweetened soda consumption			0.59	0.036
Chip consumption			0.51	0.015
CDC domain 6: physical inactivity				
Watching TV or videos			0.38	0.014
Playing video games			0.44	0.014
Using a computer			0.31	0.018
Final eigenvalues	5.08	2.01	1.13	
Cronbach’s alpha (standardized)	0.87	0.67	0.61	
McDonald’s omega	0.88	0.54	0.57	
Confirmatory factor analysis^a^: RMSEA (90% CI)	0.088 (0.086, 0.089)			
Confirmatory factor analysis^a^: SRMR	0.071			
Confirmatory factor analysis^a^: AGFI	0.807			

Exploratory factor analysis using iterated principal axis factoring and promax rotation. Factor loadings lower than 0.3 were suppressed

*CDC* Centers for Disease Control and Prevention, *RMSEA* root-mean-square error of approximation, *SRMR* standard root-mean-square residual, *AGFI* adjusted goodness-of-fit index

^a^Confirmatory factor analysis using maximum-likelihood estimation

**Table 4:** Exploratory factor analysis on risk behaviours considering all domains in the US Centers for Disease Control and Prevention risk framework in Grades 6–8 students (*N* = 5586) (Canada 2014)

Risk behaviours	Factor 1 Overt Risk Taking	Factor 2 Aversion to a Healthy Lifestyle	Factor 3 Screen Time Syndrome	Intraclass correlation
CDC domain 1: smoking cigarettes				
Lifetime smoking history	0.73			0.047
Use of alternative tobacco products	0.76			0.067
CDC domain 2: alcohol and illicit substance use				
Frequency of alcohol consumption	0.57			0.074
Lifetime drunkenness history	0.65			0.050
CDC domain 4: high-risk manifest behaviours				
Non-helmet use on a bicycle	0.35			0.149
Physical fighting	0.30			0.029
CDC domain 5: unhealthy dietary pattern				
Caffeinated energy drink consumption	0.42			0.046
CDC domain 5: unhealthy dietary pattern				
Fruit consumption		0.62		0.045
Vegetable consumption		0.61		0.053
Orange vegetable consumption		0.51		0.028
CDC domain 6: physical inactivity				
Duration of outdoor play		0.36		0.052
Participation in organized sports		0.38		0.034
Frequency of exercise outside school hours		0.48		0.029
CDC domain 5: unhealthy dietary pattern				
Sweet consumption			0.37	0.023
Sugar-sweetened soda consumption			0.50	0.066
Chip consumption			0.43	0.034
CDC domain 6: physical inactivity				
Watching TV or videos			0.50	0.029
Playing video games			0.55	0.029
Using a computer			0.50	0.043
Final eigenvalues	3.06	1.65	1.08	
Cronbach’s alpha (standardized)	0.75	0.65	0.66	
McDonald’s omega	0.68	0.63	0.63	
Confirmatory factor analysis^a^: RMSEA (90% CI)	0.074 (0.072, 0.076)			
Confirmatory factor analysis^a^: SRMR	0.067			
Confirmatory factor analysis^a^: AGFI	0.885			

Exploratory factor analysis using iterated principal axis factoring and promax rotation. Factor loadings lower than 0.3 were suppressed

*CDC* Centers for Disease Control and Prevention, *RMSEA* root-mean-square error of approximation, *SRMR* standard root-mean-square residual, *AGFI* adjusted goodness-of-fit index

^a^Confirmatory factor analysis using maximum-likelihood estimation

In both grade groups, modest correlations were observed between common factors. *Overt Risk Taking* was marginally correlated to both *Aversion to a Healthy Lifestyle* (Grades 9–10: *r* = 0.06, *r*_corrected_ = 0.08; Grades 6–8: *r* = 0.21, *r*_corrected_ = 0.30) and the *Screen Time Syndrome* (Grades 9–10: *r* = 0.14, *r*_corrected_ = 0.19; Grades 6–8: *r* = 0.21, *r*_corrected_ = 0.32). There were positive correlations of moderate strength between the *Screen Time Syndrome* and *Aversion to a Healthy Lifestyle* in both grade groups (Grades 9–10: *r* = 0.30, *r*_corrected_ = 0.47; Grades 6–8: *r* = 0.31, *r*_corrected_ = 0.47). However, overall these correlations suggest that these three factors are distinct from one another. Intraclass correlations (ICC) calculated for each risk behaviour were variable and suggested low-to-moderate variance attributable to the school level (Grades 9–10: ICC range = 0.011–0.096, Table 3; Grades 6–8: ICC range = 0.023–0.149, Table 4).

Confirmatory factor analyses suggested that the Grades 9–10 (SRMR = 0.071, RMSEA = 0.088, AGFI = 0.807, *N* = 3693; *χ* ^2^ = 6660, *df* = 227, *p* < 0.001) and the Grades 6–8 final models (SRMR = 0.067, RMSEA = 0.074, AGFI = 0.885, *N* = 5984; *χ* ^2^ = 4995, *df* = 149, *p* < 0.001) had modest fits (Hooper et al. 2008).

Confirmatory factor analyses were also performed to investigate a two-factor model that combined items from the *Aversion to a Healthy Lifestyle* and *Screen Time Syndrome* categories. This two-factor model performed poorer across all of the fit indices (Grades 9–10: SRMR = 0.079, RMSEA = 0.094, AGFI = 0.782; Grades 6–8: SRMR = 0.083, RMSEA = 0.094, AGFI = 0.812) when compared to the three-factor model.

## Discussion

This study provides a contemporary and comprehensive examination of risk-taking behaviours among Canadian adolescents—a population that is vital to examine in the field of multiple risk behaviour as it is a critical period of the life course. The objective of this study was to evaluate relationships between risk behaviours, both novel and contemporary, derived from a list inspired by the diverse domains of the CDC framework. We conducted this analysis within a Canadian context. Based on that objective, we found that three composite indicators of clustered risk behaviour emerged from our analysis. Interestingly, these new latent constructs encompassed behaviours that crossed all six domains of risk described within the CDC framework. Our findings were fairly consistent across two broad developmental periods (Grades 6–8 and 9–10), although the items available to measure adolescent risk-taking were limited in the youngest age group.

The CDC’s Youth Risk Behaviour Survey of adolescent risk behaviour evaluates and monitors the domains of behaviour most closely associated with known leading causes of morbidity and mortality (Kann et al. 2016). Continuous revision based on public health data and expert opinion means that it consistently captures a comprehensive list of new and long-standing risk behaviours that impact youth, which makes it a frequently used tool for prevention and harm-reduction programs. However, many current public health programs remain outdated in their use of the CDC framework by targeting individual domains of behaviour (i.e., alcohol and illicit substance use), or even individual risk behaviours within a domain (i.e., alcohol consumption) for public health interventions and ignore the well-established concept of their inter-related and clustered natures. By incorporating lessons of Problem Behaviour Theory (Jessor 1991, 2014), our study uses the comprehensive CDC risk domains to identify three clusters of risk behaviour that may provide more focused public health interventions targeting contemporary populations of young Canadians.

Our approach to conceptualizing risk behaviour recognizes the complex relationships that exist amongst them, as well as their possible joint effects on disease etiology. The items indicated by the CDC domains are intimately related in interpretable and potentially unexpected ways, consistent across grade groups. As a result, these behaviours should be observed and measured collectively under each of the three categories to be properly understood and managed.

The three risk categories that were identified by factor analysis incorporated items that are well recognized within the adolescent health research literature. The *Overt Risk Taking* category largely encompasses behaviours found in the traditional adolescent risk studies (Maggs et al. 1997; de Looze et al. 2012). However, we believe that the benefits of our analysis lie in the more contemporary expressions of risk-taking that were incorporated. For example, the inclusion of *caffeinated energy drinks* and *alternative tobacco products* highlights emergent areas of related risk that reveal either the true breadth of this category, or behaviours associated with more moderate risk tolerance that indicate early development of risk-taking in this domain. In contrast, the traditional public health programs targeting individual behaviours, such as cigarette use, may forego the opportunity to educate and prevent other behaviours within the same category that an adolescent is likely already participating in (such as consumption of caffeinated energy drinks, alternative tobacco products, cannabis use, and riding a bicycle without a helmet). The *Screen Time Syndrome* and *Aversion to a Healthy Lifestyle* categories indicated close relationships between diet, physical activity, and sedentary behaviour that have been recognized in the past research (Leech et al. 2014). Nevertheless, our empirical distinction between these categories also supports studies that show that a lack of moderate-to-vigorous physical activity and excessive screen time represent separate and distinct behaviours among adolescent populations (Pearson et al. 2014; Brindova et al. 2015). Our study’s risk categories represent measures of three separate types of adolescent risk behaviour that were found to be robust through confirmatory analysis in a separate subset of our study population.

Although psychometric research on the relationships amongst risk behaviours has been conducted in the past, none have used an established framework to ensure that they have captured a group of behaviours that are associated with the current leading causes of illness and injury among youths. Our three categories incorporate behaviours that span each of the six of the CDC risk behaviour domains, and the resultant composite scores are, perhaps, more consistent with the way that adolescents behave socially compared to other studies of multiple risk behaviours. These three risk categories have implications for the development and targeting of public health interventions that improve upon individual risk behaviour approaches, and can broaden clustered risk behaviour approaches with a narrow scope (i.e., Sloboda et al. 2009).

Of importance, our analysis extends the existing research (e.g., Pickett et al. 2002; De La Haye et al. 2014) by being amongst the first studies to factor analytically derive composite indicators of risk-taking based on an established framework. This study identified three ways that adolescents engage in risk-taking behaviours—each presumably having their own upstream determinants and downstream health consequences. Further research is now needed to confirm these as stable and consistent composite indicators of risk-taking in other study populations and contexts, and to evaluate the health outcomes and risk factors associated with each distinct category. Future intervention programs could then target the risk factors of each category to address their associated negative health outcomes (Jackson et al. 2012; Hale et al. 2014).

Admittedly, research such as this is often limited by its reliance on self-reported data. The HBSC attempts to minimize this limitation through the emphasis of confidentiality of responses (Currie et al. 2012). Nevertheless, students may not have answered truthfully to all the questions due to social desirability biases. Similarly, risk behaviour and sensitive questions have higher rates of non-response, most notably those surrounding sexual behaviour. Several of the items in the HBSC ask about days of lifetime exposure to specific risk behaviours and may misclassify newly emergent high-frequency engagement as moderate engagement. Finally, the CDC risk framework that inspired our work may not be completely applicable to the Canadian HBSC study population, based on cultural and age differences. Finally, we reported the intraclass correlation for each risk behaviour included in our composite measures and note that some behaviours showed moderate clustering effects at the school level. Although the majority of the risk behaviours in our study had negligible clustering effects at the school level, this analysis did not account for such clustering and may have overestimated variance at the student-level. Based on our reported measures, future analyses may choose to account for school-level clustering effects.

### Conclusion

This study used a large sample of Canadian adolescents to evaluate relationships amongst adolescent risk behaviours. This psychometric research was inspired by the six-domain framework outlined by the CDC (Kann et al. 2016). Our empirical analysis, which included both exploratory and confirmatory factor analytic techniques, found that adolescent risk behaviours cluster in predictable patterns crossing the different CDC risk domains. Three categories of risk behaviours emerged based on the six-domain framework: (1) *Overt Risk Taking*, (2) *Aversion to a Healthy Lifestyle*, and (3) *Screen Time Syndrome.* These categories build on the existing studies of multiple risk behaviour, and inform research and intervention efforts aimed at preventing adolescent illness and injury. Future research could use this new framework of adolescent risk behaviour to study their upstream determinants, as well as their joint causes of negative health outcomes.

## References

[CR1] Basen-Engquist K, Edmundson EW, Parcel GS (1996). Structure of health risk behavior among high school students. J Consult Clin Psychol.

[CR2] Brener ND, Kann L, Kinchen SA (2004). Methodology of the youth risk behavior surveillance system. Morb Mortal Wkly Rep.

[CR3] Brindova D, Veselska ZD, Klein D (2015). Is the association between screen-based behaviour and health complaints among adolescents moderated by physical activity?. Int J Public Health.

[CR4] Currie C, Zanotti C, Morgan A, et al (2012) Social determinants of health and well-being among young people. Health Behaviour in School-aged Children (HBSC) study: international report from the 2009/2010 survey. World Health Organization. http://www.euro.who.int/en/publications/abstracts/social-determinants-of-health-and-well-being-among-young-people.-health-behaviour-in-school-aged-children-hbsc-study. Accessed 9 Feb 2017

[CR5] De La Haye K, D’Amico EJ, Miles JNV (2014). Covariance among multiple health risk behaviors in adolescents. PLoS ONE.

[CR6] de Looze M, van den Eijnden R, Verdurmen J (2012). Parenting practices and adolescent risk behavior: rules on smoking and drinking also predict cannabis use and early sexual debut. Prev Sci.

[CR7] Fabrigar LR, Fabrigar LR, Wegener DT (1999). Evaluating the use of exploratory factor analysis in psychological research. Psychol Methods.

[CR8] Freeman J, King M, Pickett W (2016) Health behaviour in school-aged children (HBSC) in Canada: focus on relationships. Public Health Agency of Canada. http://healthycanadians.gc.ca/publications/science-research-sciences-recherches/health-behaviour-children-canada-2015-comportements-sante-jeunes/index-eng.php. Accessed 18 Feb 2017

[CR9] Goniewicz ML, Leigh NJ, Gawron M (2016). Dual use of electronic and tobacco cigarettes among adolescents: a cross-sectional study in Poland. Int J Public Health.

[CR10] Hale DR, Fitzgerald-Yau N, Viner RM (2014). A systematic review of effective interventions for reducing multiple health risk behaviors in adolescence. Am J Public Health.

[CR11] Hooper D, Coughlan J, Mullen M (2008). Structural equation modeling: guidelines for determining model fit. Electron J Bus Res Methods.

[CR12] Jackson C, Geddes R, Haw S, Frank J (2012). Interventions to prevent substance use and risky sexual behaviour in young people: a systematic review. Addiction.

[CR13] Jessor R (1991). Risk behavior in adolescence: a psychosocial framework for understanding and action. J Adolesc Heal.

[CR14] Jessor R, Lerner RM, Petersen AC, Silbereisen RK, Brooks-Gunn J (2014). Problem Behavior Theory: a half-century of research on adolescent behavior and development. The developmental science of adolescence: history through autobiography.

[CR15] Kabacoff RI (2003) Determining the dimensionality of data: a SAS macro for parallel analysis coders’ corner. In: Proceedings of the twenty-eighth annual SAS users group international conference. pp 1–3

[CR16] Kann L, McManus T, Harris WA (2016). Youth risk behavior surveillance—United States, 2015. Morb Mortal Wkly Rep.

[CR17] Kline P (1994). An easy guide to factor analysis.

[CR18] Kline RB, Petscher Y, Schatschneider C, Compton DL (2013). Exploratory and CONfiRMATORY FACTOR ANALYSIS. Applied quantitative analysis in education and the social sciences.

[CR19] Leech RM, McNaughton SA, Timperio A (2014). The clustering of diet, physical activity and sedentary behavior in children and adolescents: a review. Int J Behav Nutr Phys Act.

[CR20] Lindberg LD, Boggess S, Williams S (1995) Multiple threats: the co-occurrence of teen health risk behaviors. Washington, DC

[CR21] Lytle L (2002). Nutritional issues for adolescence. J Am Diet Assoc.

[CR22] Maggs JL, Frome PM, Eccles JS, Barber BL (1997). Psychosocial resources, adolescent risk behaviour and young adult adjustment: is risk taking more dangerous for some than others?. J Adolesc.

[CR23] Pearson N, Braithwaite RE, Biddle SJH (2014). Associations between sedentary behaviour and physical activity in children and adolescents: a meta-analysis. Obes Rev.

[CR24] Pérez Fuentes MDC, del Molero Jurado MM, Carrión Martínez JJ (2016). Sensation-seeking and impulsivity as predictors of reactive and proactive aggression in adolescents. Front Psychol.

[CR25] Pfortner TK, De Clercq B, Lenzi M (2015). Does the association between different dimension of social capital and adolescent smoking vary by socioeconomic status? a pooled cross-national analysis. Int J Public Health.

[CR26] Pickett W, Schmid H, Boyce WF (2002). Multiple risk behavior and injury: an international analysis of young people. Arch Pediatr Adolesc Med.

[CR27] Riesch SK, Kedrowski K, Brown RL (2013). Health-risk behaviors among a sample of US pre-adolescents: types, frequency, and predictive factors. Int J Nurs Stud.

[CR28] Schane RE, Ling PM, Glantz SA (2010). Health effects of light and intermittent smoking: a review. Circulation.

[CR29] Schmitt N (1996). Uses and abuses of coefficient alpha. Psychol Assess.

[CR30] Seifert SM, Schaechter JL, Hershorin ER, Lipshultz SE (2011). Health effects of energy drinks on children, adolescents, and young adults. Pediatrics.

[CR31] Sloboda Z, Stephens RC, Stephens PC (2009). The adolescent substance abuse prevention study: a randomized field trial of a universal substance abuse prevention program. Drug Alcohol Depend.

[CR32] Spring B, Moller AC, Coons MJ (2012). Multiple health behaviours: overview and implications. J Public Health (Bangkok).

[CR33] Thompson K, Stockwell T, Leadbeater B, Homel J (2014). Association among different measures of alcohol use across adolescence and emerging adulthood. Addiction.

[CR34] Turner C, McClure R, Pirozzo S (2004). Injury and risk-taking behavior: a systematic review. Accid Anal Prev.

[CR35] Van Pelt DN, Clemens J, Brown B, Palacios M (2015) Where Our Students Are Educated: Measuring student enrolment in Canada. Fraser Institute. https://www.fraserinstitute.org/studies/where-our-students-are-educated-measuring-student-enrolment-in-canada. Accessed 08 Mar 2017

[CR36] Yarber WL, Parrillo AV (1992). Adolescents and sexually transmitted diseases. J Sch Health.

[CR37] Zhang Z, Yuan K (2015) Robust coefficient alpha and omega with missing and non-normal data. R package version 0.5-23.1997

